# Evaluation of a Structured, Iteratively Developed Surgical and Perioperative Care Teaching Programme for Undergraduate Medical Students: A Single-Centre Educational Evaluation

**DOI:** 10.7759/cureus.112874

**Published:** 2026-07-17

**Authors:** Jawad Ahmad, Amun Mahmood, Abigail Smith, Gursharun Hayer

**Affiliations:** 1 Education, Sandwell and West Birmingham NHS Trust, West Bromwich, GBR; 2 Medical Education and Simulation, Sandwell and West Birmingham NHS Trust, West Bromwich, GBR

**Keywords:** clinical teaching, curriculum mapping, formative assessment, medical students, mock osce, perioperative care, quality improvement, surgical education, undergraduate medical education

## Abstract

Background

Undergraduate surgical education is often affected by variable clinical exposure, inconsistent teaching opportunities, and limited structured preparation for peri-operative care and surgical assessments. Year 4 medical students undertaking the Surgery and Peri-operative Care module at Sandwell Hospital reported low confidence across several surgical and peri-operative domains.

Methods

A single-centre educational evaluation was conducted at Sandwell and West Birmingham NHS Trust (SWBH) among Year 4 medical students undertaking the University of Birmingham Surgery and Peri-operative Care (SPC) attachment. "SPC Made Easy" was designed by the Clinical Teaching Fellows using University learning outcomes and delivered across five-week rotations as six two-hour sessions. Teaching was delivered by resident doctors and the Clinical Teaching Fellows and covered key SPC topics, including Ear, Nose and Throat (ENT), anaesthetics, urology, breast surgery, orthopaedics, and peri-operative care. The programme was iteratively refined using student feedback: early quiz-style consolidation was removed after students reported limited educational value, and practical clinical skills teaching using models and mannequins was incorporated, including digital rectal, breast, and testicular examinations. The programme also included case-based learning and a supervised peer-led mock Objective Structured Clinical Examination (OSCE) with post-station feedback. Anonymous pre- and post-programme questionnaires assessed self-rated knowledge across surgical and peri-operative domains, while separate pre- and post-mock OSCE questionnaires assessed confidence in OSCE-related tasks. Because responses were anonymous and could not be paired, data were analysed as independent samples using descriptive statistics and Mann-Whitney U tests.

Results

A total of 43 pre-programme and 20 post-programme questionnaires were returned. Self-rated knowledge improved across all seven surgical and peri-operative domains, with statistically significant improvements in anaesthetics, pre-operative care, ENT surgery, urology, orthopaedic surgery, rheumatology, and post-operative care. Median ratings increased from 1-2 before the programme to 4-5 after the programme across most domains. The largest improvements were seen in ENT, urology, orthopaedics, and post-operative care. In the mock OSCE evaluation, confidence improved across all assessed domains, including overall OSCE performance, surgical history taking, practical examination skills, and giving peer feedback. Post-programme acceptability was high, with all respondents stating that they would recommend SPC Made Easy to future students.

Conclusions

SPC Made Easy was associated with improved self-rated knowledge and confidence across core surgical and peri-operative domains. The programme was feasible to deliver within a five-week clinical attachment and was strengthened by iterative student feedback, which shifted the programme from passive quiz-style consolidation towards practical, clinically relevant examination skills. The findings are limited by the single-centre design, small post-programme and mock OSCE sample sizes, anonymous unmatched responses, and reliance on self-reported outcomes rather than objective performance measures.

## Introduction

Undergraduate surgical education is frequently affected by variability in clinical exposure, teaching structure, and opportunities for practical skills development. This is particularly important because surgical placements often depend heavily on opportunistic learning in clinics, theatres, and ward environments, rather than on consistently delivered curriculum-mapped teaching. A national undergraduate assessment by Lee MJ et al. raised concerns about the adequacy of surgical training in medical school, while Davis CR et al. demonstrated variation in surgical and procedural skills training across UK medical schools [1,2]. More recently, a systematic review of undergraduate surgical skills teaching in the UK concluded that there remains a clear deficit in surgical skills teaching, with a need for structured approaches such as simulation, near-peer teaching, and practical skills sessions [3].

The operating theatre and peri-operative environment can provide valuable learning opportunities, but they are not always reliable educational settings for undergraduate students. Students may be limited by unclear learning objectives, variable supervision, service pressures, and uncertainty about how to participate meaningfully in the clinical environment. Croghan SM et al. described the operating theatre as a potentially valuable but challenging classroom for medical students, highlighting the need for clearer structure and better educational support within surgical placements [4]. These findings are relevant to Surgery and Peri-operative Care (SPC) teaching, where students are expected to develop not only factual knowledge but also confidence in surgical assessment, peri-operative decision-making, and practical examination skills.

Low confidence and dissatisfaction with surgical teaching have also been reported in studies evaluating undergraduate surgical skills interventions. Hakim MA et al. reported that UK medical students described poor satisfaction with surgical teaching and argued that surgical skills workshops should be incorporated into the undergraduate curriculum [5]. Similarly, Mughal Z et al. developed a national undergraduate surgical workshop focused on recognition and management of the acutely unwell surgical patient, noting that this was an area in which medical students had limited exposure and that structured workshop-based teaching improved confidence [6]. Together, these studies support the view that undergraduate surgical education benefits from structured, practical, and learner-centred interventions rather than reliance on clinical exposure alone.

A similar educational gap was identified locally at Sandwell and West Birmingham NHS Trust (SWBH) through an anonymous retrospective needs assessment of Year 5 students who had previously completed the Year 4 SPC attachment. Seventeen students responded to categorical and Likert-style questions. Of these, 14 of 17 respondents reported that they had been either “not at all confident” or “not very confident” in their understanding of SPC before the attachment, while 15 of 17 stated that they would have benefited from a structured teaching programme. These findings supported the wider published literature and suggested that the local problem was not just dissatisfaction with one placement, but part of a broader pattern of variability in undergraduate surgical education.

In response to this identified gap, SPC Made Easy was developed as a structured, curriculum-aligned teaching programme for Year 4 medical students undertaking the SPC attachment.

The primary objective of this evaluation was to assess whether SPC Made Easy was associated with changes in students’ self-rated knowledge and confidence across core surgical and peri-operative domains through structured teaching, case-based discussion, practical clinical skills teaching, and mock Objective Structured Clinical Examination (OSCE) preparation.

The secondary objectives were to evaluate students’ perceived preparedness for OSCE-related tasks, assess programme acceptability, and describe the feasibility of delivering an iteratively developed undergraduate surgical teaching programme within a district general hospital placement.

## Materials and methods

Study design and setting

This was a single-centre educational evaluation conducted at SWBH, United Kingdom. The evaluation focused on Year 4 medical students undertaking the University of Birmingham SPC attachment.

The SPC module is designed by the University of Birmingham and delivered over a five-week clinical attachment. Students rotate through surgical specialties, including Ear, Nose and Throat (ENT), orthopaedics, urology, anaesthetics, and breast surgery. Students are provided with learning outcomes in their rotation booklets, which guide the knowledge, clinical reasoning, and practical skills expected during the module.

The programme was delivered as part of local undergraduate teaching provision. Attendance at scheduled teaching was part of the educational programme, but completion of questionnaires was voluntary and anonymous.

Participants

Participants were Year 4 medical students undertaking the SPC attachment at SWBH. Students rotated through the placement in five-week blocks. Since the student groups changed at the end of each rotation, the programme had to be delivered within a short timeframe, and feedback collection was time-limited.

Inclusion and exclusion criteria

For the retrospective needs assessment, the inclusion criteria were University of Birmingham Year 5 medical students placed at SWBH who had previously completed the SPC attachment during Year 4, regardless of the hospital site at which they completed that attachment. Students were excluded if they were not Year 5 University of Birmingham medical students, had not previously completed the SPC attachment, or did not complete the anonymous questionnaire.

For the programme evaluation, the inclusion criteria were University of Birmingham Year 4 medical students undertaking the SPC attachment at SWBH during the evaluation period. Students were excluded from the programme evaluation dataset if they were not undertaking the Year 4 SPC attachment at SWBH, were from another year group or placement site, or did not complete the relevant anonymous questionnaire.

For the mock OSCE evaluation, students were included if they participated in the peer-led mock surgical OSCE during the SPC attachment and completed the anonymous pre- or post-session confidence questionnaire. Students who did not attend the mock OSCE or did not complete the questionnaire were excluded from that specific analysis.

Retrospective needs assessment

A retrospective anonymous needs assessment was distributed to Year 5 medical students who had previously completed the SPC attachment. Seventeen students responded. The questionnaire explored previous experience of SPC teaching, perceived confidence in peri-operative care, satisfaction with the structure of surgical teaching, and perceived need for a more organised curriculum-aligned programme. The questionnaire included categorical and Likert-style items, with opportunities for free-text feedback. The full retrospective needs assessment questionnaire is provided in Appendix 1.

The needs assessment identified low student confidence in peri-operative care and dissatisfaction with the structure and consistency of previous surgical teaching. These findings supported the development of SPC Made Easy as a structured intervention targeting a local educational gap.

Intervention

SPC Made Easy was designed by the Clinical Teaching Fellow as a structured, curriculum-aligned teaching programme for Year 4 medical students undertaking the SPC attachment at SWBH. The programme was mapped to the University of Birmingham SPC learning outcomes to ensure that teaching content aligned with the required curriculum and was appropriate for students’ stage of training (Table 1). Any subsequent changes to the programme were also mapped back to the curriculum to ensure continued relevance and educational consistency.

**Table 1:** Overview of the Year 4 Surgery and Peri-operative Care curriculum. The Surgery and Peri-operative Care curriculum covers core surgical knowledge, peri-operative principles, specialty-specific presentations, and practical examination skills expected of Year 4 medical students. ENT: Ear, Nose, and Throat; OSCE: Objective Structured Clinical Examination.

Curriculum area	Core content covered
Common surgical presentations	Recognition and initial assessment of common surgical conditions, including acute presentations and red-flag symptoms.
Peri-operative care	Principles of pre-operative assessment, optimisation, consent, peri-operative risk, and post-operative recovery.
Anaesthetics	Basic anaesthetic principles, peri-operative safety, risk assessment, and the role of anaesthesia in surgical care.
ENT	Assessment and management principles for common ear, nose, and throat presentations.
Urology	Assessment of common urological presentations, urinary symptoms, acute urological problems, and relevant examination principles.
Orthopaedics	Assessment of common musculoskeletal presentations, fracture assessment, and basic orthopaedic management principles.
Breast surgery	Assessment of breast symptoms, breast examination, and principles of referral and management.
Practical examination skills	Breast examination, testicular examination, and digital rectal examination.
Post-operative complications	Recognition, initial management, and escalation of common post-operative complications.
OSCE preparation	Application of surgical knowledge, history taking, examination, and clinical reasoning in assessment-style scenarios.

The programme was implemented across five consecutive Year 4 SPC blocks. Each block lasted five weeks and included approximately 10 students. Teaching was delivered weekly and consisted of six sessions, each lasting approximately two hours. Sessions were delivered by resident doctors with an interest in surgery and by the Clinical Teaching Fellow. The Clinical Teaching Fellow coordinated the programme, oversaw delivery, and met regularly with resident doctors to maintain consistency, share feedback, and ensure that teaching remained aligned with the intended learning outcomes.

The programme covered key areas of the SPC attachment, including ENT, anaesthetics, urology, breast surgery, orthopaedics, and broader peri-operative care themes. Each session combined curriculum-mapped teaching, case-based discussion, and opportunities for applied learning. The aim was to move beyond passive revision and provide students with structured preparation for clinical placement learning, surgical assessment, and OSCE-style tasks.

The programme evolved through iterative feedback across the five-week rotations. In the first cycle, a protected weekly teaching programme was introduced to address the absence of formal surgical teaching within the block. The first version included structured weekly teaching delivered by Clinical Teaching Fellows and resident doctors, with anonymous pre-block and post-block questionnaires used to measure confidence and perceived preparedness. Early feedback suggested that students valued regular structured teaching, but attendance was initially affected because some students understood the sessions to be optional.

In the second cycle, attendance expectations were clarified with the university, and the sessions were made compulsory as part of the local teaching programme. In response to student feedback, practical clinical examination teaching was added, including digital rectal examination (DRE), breast examination, and testicular examination. Students felt that the programme required more direct clinical and assessment relevance, and these practical components were therefore introduced to strengthen the link between the teaching programme, clinical placement learning, and OSCE preparation.

In the third cycle, quiz-style formative consolidation was reduced and then removed because students reported that it did not add sufficient educational value. Instead, the programme placed greater emphasis on clinical application and practical examination skills. Practical sessions were delivered using appropriate clinical models and mannequins. For example, DRE teaching was delivered using a DRE model. Resident doctors and the Clinical Teaching Fellow used relevant models to demonstrate technique, discuss examination structure, and support students in understanding how these skills should be applied safely and professionally in clinical and OSCE settings.

The final programme therefore consisted of curriculum-mapped teaching, case-based learning, practical clinical examination skills teaching, and a peer-led mock surgical OSCE. The mock OSCE was designed to provide students with low-stakes exposure to assessment-style tasks and was self-run by students but supervised by the resident doctors and Clinical Teaching Fellow delivering SPC Made Easy. After the mock OSCE, faculty reviewed each station with the students and provided feedback on clinical reasoning, communication, examination structure, and how to approach similar stations in future assessments.

Across all cycles, the programme remained centrally coordinated by the Clinical Teaching Fellow to maintain curriculum alignment and consistency of delivery. The main lessons from the implementation process were that protected teaching time improved engagement when attendance expectations were clear, that students valued practical examination content, and that mock assessment practice was perceived as more useful than quiz-based revision. Post-block data collection remained challenging because students frequently moved to other hospital sites after completing the five-week block.

Data collection

Four sources of data were used. First, an anonymous retrospective Year 5 needs assessment was used to establish the baseline educational problem among students who had previously completed the SPC attachment. This questionnaire used categorical and Likert-style items and did not collect identifiable data.

Second, a pre-programme questionnaire was distributed to Year 4 students before the teaching programme. This assessed baseline self-rated knowledge across surgical and peri-operative domains, as well as preferred learning styles and areas of low confidence.

Third, a post-programme questionnaire was distributed after completion of the teaching programme. This assessed self-rated knowledge across the same domains, as well as acceptability, perceived exam preparedness, and perceived relevance to clinical decision-making. The programme-level pre- and post-programme questionnaire items are provided in Appendix 2.

Fourth, a separate mock OSCE questionnaire was administered immediately before and after the peer-led mock OSCE. This assessed confidence in overall OSCE performance, surgical history taking, practical examination skills, and giving peer feedback as an examiner. All questionnaires were anonymous. No identifiable student data were collected. Because no matching identifier was used, individual pre- and post-programme responses could not be linked. The pre- and post-mock OSCE questionnaire items are provided in Appendix 3.

Outcome measures

The primary outcome was change in self-rated knowledge across surgical and peri-operative domains. These domains included anaesthetics, pre-operative care, ENT surgery, urological surgery, orthopaedic surgery, rheumatology, and post-operative care.

Secondary outcomes included self-rated confidence in mock OSCE-related tasks and post-programme acceptability, including perceived exam preparedness and willingness to recommend the programme.

The outcomes were self-reported and should therefore be interpreted as measures of perceived knowledge, confidence, and preparedness rather than objective clinical competence.

Sample size

This was a pragmatic educational evaluation of a local teaching programme. A formal prospective sample-size calculation was not performed. All eligible students who attended the relevant attachment during the evaluation period were invited to complete the anonymous questionnaires. The final sample size was therefore determined by student placement numbers and voluntary questionnaire completion.

Statistical analysis

Pre- and post-programme questionnaire responses were analysed as independent samples because responses were anonymous and could not be matched at the individual level. Likert-scale responses were summarised using medians and interquartile ranges, with mean differences also reported to describe the magnitude of change.

The Mann-Whitney U test was used to compare pre- and post-programme responses. Standardised effect size r was reported for each comparison. Two-sided p-values of less than 0.05 were considered statistically significant.

Given the small sample size in the mock OSCE evaluation, these findings were interpreted cautiously and used primarily to describe directional changes in self-reported confidence.

Ethical considerations

This project was conducted as an educational evaluation of a local teaching programme. It was registered with and approved by the local Clinical Effectiveness Team at SWBH under audit registration numbers 3105.1 and 3271.1. All questionnaire responses were anonymous and voluntary. No patient data or identifiable student data were collected. According to local institutional guidance for educational evaluation and quality improvement activity, formal research ethics committee approval was not required.

## Results

Programme-level questionnaire responses

A total of 43 pre-programme and 20 post-programme questionnaires were returned by Year 4 medical students undertaking the SPC attachment. Because responses were anonymous and could not be linked at the individual level, pre- and post-programme data were treated as independent samples.

Self-Rated Knowledge Across Surgical and Peri-operative Domains

Across all seven surgical and peri-operative domains, the post-programme group reported higher self-rated knowledge than the pre-programme group (Table 2). Median ratings increased from 1-2 before the programme to 4-5 after the programme across most domains. All comparisons were statistically significant (p < 0.001).

**Table 2:** Self-rated knowledge across surgical and peri-operative domains before and after SPC Made Easy. Likert scale: 1 = minimal knowledge, 5 = excellent knowledge. The mean difference refers to the post-programme mean minus the pre-programme mean. ENT: Ear, Nose, and Throat; SPC: Surgery and Peri-operative Care.

Domain	Pre-programme median (IQR) (n = 43)	Post-programme median (IQR) (n = 20)	Mean difference	p-value	Effect size (r)
Anaesthetics	2 (1-3)	4 (4-5)	2.43	<0.001	0.74
Pre-operative care	2 (1-2)	4 (4-5)	2.38	<0.001	0.74
ENT surgery	1 (1-2)	5 (4-5)	2.68	<0.001	0.77
Urological surgery	1 (1-2)	5 (4-5)	2.66	<0.001	0.75
Orthopaedic surgery	1 (1-2)	5 (4-5)	2.70	<0.001	0.76
Rheumatology	2 (1-3)	4 (3-5)	1.54	<0.001	0.47
Post-operative care	2 (1-2)	4 (4-5)	2.42	<0.001	0.77

The largest mean increases were observed in orthopaedic surgery (+2.70), ENT surgery (+2.68), urological surgery (+2.66), anaesthetics (+2.43), and post-operative care (+2.42). Rheumatology showed the smallest mean increase (+1.54) and the greatest residual spread of post-programme responses.

These findings suggest that SPC Made Easy was associated with improved self-rated knowledge across the curriculum domains covered by the teaching programme. The consistency of improvement across domains supports the educational value of a structured, curriculum-mapped intervention.

Confidence in the Peer-Led Mock Surgical OSCE

A smaller subset of students completed questionnaires immediately before and after the peer-led mock surgical OSCE. Ten students completed the pre-mock OSCE questionnaire, and seven completed the post-mock OSCE questionnaire.

Self-rated confidence improved across all assessed OSCE-related domains (Table 3). Confidence in performing in an OSCE increased from a median of 2 before the session to 4 after the session. Confidence also improved in surgical history taking, practical examination skills, and giving peer feedback as an examiner.

**Table 3:** Self-rated confidence before and after the peer-led mock surgical OSCE. Self-rated confidence was assessed using a 5-point Likert scale, where 1 = very low confidence and 5 = very high confidence. OSCE: Objective Structured Clinical Examination; DRE: Digital rectal examination.

Confidence domain	Pre-mock OSCE median (n = 10)	Post-mock OSCE median (n = 7)	p-value	Effect size (r)
Performing in an OSCE	2	4	0.001	0.84
Surgical history taking	2	4	0.001	0.80
Surgical skills, including digital rectal examination (DRE) and testicular examination	2	4	0.002	0.74
Giving peer feedback as an examiner	3	4	0.012	0.61

Although the sample size was small, the direction of change was consistent across all assessed domains. These findings suggest that the mock OSCE may have improved students’ perceived preparedness for surgical assessment tasks, particularly practical examination skills and overall OSCE performance.

Programme Acceptability and Perceived Exam Preparedness

Post-programme feedback suggested that students were highly receptive to SPC Made Easy as an overall teaching programme (Table 4). All respondents stated that they would recommend the programme to future students. Students also reported high levels of perceived exam preparedness, improved confidence for OSCEs and multiple-choice questions (MCQs), and high engagement with the weekly structure.

**Table 4:** Post-programme evaluation of acceptability and perceived exam preparedness. A 5-point Likert scale was used, where 1 = the lowest level of agreement, confidence, preparedness, or satisfaction, and 5 = the highest level. Yes/No items are reported as the percentage of respondents who answered “Yes.” The value of n represents the number of responses for each individual evaluation item. SPC: Surgery and Peri-operative Care; OSCEs: Objective Structured Clinical Examinations; MCQs: Multiple-choice questions.

Evaluation item	Result	Response scale and n
Would recommend SPC Made Easy	100% Yes	Yes/No, n = 20
Better prepared for Year 4 exams and OSCEs	4.70 (0.73)	1-5, n = 20
Weekly structure aided engagement	4.70 (0.57)	1-5, n = 20
More confident for OSCEs after the programme	4.67 (0.59)	1-5, n = 18
More confident for MCQs after the programme	4.67 (0.49)	1-5, n = 18
Perceived impact on clinical decision-making	88% Yes	Yes/No, n = 17

Importantly, the programme’s acceptability should be interpreted in the context of its iterative development. Although the earliest version included a quiz-style activity, feedback from the first rotation indicated that students did not find this component as useful as intended. The programme was therefore modified to prioritise practical clinical examination skills and assessment-relevant teaching. The positive post-programme feedback is therefore best understood as support for the evolved SPC Made Easy programme, rather than as endorsement of the original quiz-based format.

Overall, the programme was associated with high acceptability and improved perceived preparedness. The iterative replacement of quiz-based activity with practical examination teaching appears to have strengthened the relevance of the programme to students’ clinical and assessment needs.

## Discussion

This evaluation found improvements in self-rated knowledge across all measured surgical and peri-operative domains following implementation of SPC Made Easy. The largest gains were seen in orthopaedic surgery, ENT, urology, and post-operative care, which were areas where students reported low baseline confidence. This pattern suggests that a short five-week intervention can address perceived gaps in preparedness when teaching is deliberately mapped to curriculum outcomes and targeted to areas of student need.

The most important educational message from this project is not simply that structured teaching was helpful, but that the programme became more effective because it responded to learner feedback and shifted from passive quiz-style consolidation to practical, assessment-relevant clinical skills teaching. The first cohort did not perceive the quiz component as adding sufficient value. In response, the programme was redesigned to prioritise practical examination teaching using models and mannequins. This made the intervention more relevant to the clinical and OSCE tasks students were expected to perform.

This shift towards practical clinical skills aligns with experiential learning principles, where learners benefit from active participation, rehearsal, feedback, and reflection [7]. It is also supported by evidence from undergraduate surgical skills teaching, which suggests that practical and simulation-based approaches can improve student engagement, confidence, and preparedness [5,6,8]. In this study, the addition of digital rectal, breast, and testicular examination teaching helped address a gap between clinical exposure and assessment preparation, particularly for skills that students may have limited opportunities to practise during routine placements.

The mock OSCE component further extended the programme from knowledge acquisition into clinical performance rehearsal. Students were able to practise surgical history taking, practical examination structure, and peer feedback in a low-stakes environment. OSCE-style assessment is well established as a structured method for assessing clinical competence, and in this programme, it was used formatively to support rehearsal and feedback rather than summative judgement [9]. Although the mock OSCE sample was small, the direction of change across all confidence domains suggests that this component was valued by students and may have improved perceived assessment preparedness.

The findings should be interpreted cautiously because the study measured self-rated knowledge and confidence rather than objective performance. Increased confidence does not necessarily equate to improved competence, and previous surgical education research has shown that confidence and competence should not be assumed to be interchangeable [10]. Nevertheless, confidence remains educationally relevant in clinical placements, where students who feel underprepared may be less likely to participate actively, volunteer for supervised practice, or seek feedback. Improving confidence may therefore support engagement, even though competence requires separate objective assessment.

Overall, the findings suggest that SPC Made Easy was associated with improved self-rated knowledge, confidence, and perceived preparedness among Year 4 medical students. However, because this was a single-centre educational evaluation using anonymous unmatched questionnaires, the results should be interpreted cautiously. The data support improvement in perceived preparedness at the group level, but they do not demonstrate individual-level improvement or objective improvement in clinical competence.

The evaluation also suggests that a structured, practical, and curriculum-aligned surgical teaching programme can be delivered feasibly within a district general hospital placement using available teaching resources. Its delivery by resident doctors and a Clinical Teaching Fellow indicates that resource-limited teaching teams may be able to provide effective undergraduate surgical education when teaching is organised, mapped to curriculum outcomes, and responsive to learner feedback. Future cycles should strengthen the evaluation design by using matched anonymous identifiers, protected time for questionnaire completion, and objective assessment outcomes to determine whether improved confidence translates into measurable performance gains.

Limitations

This evaluation has several limitations that affect the strength and interpretation of the findings.

First, the study relied on self-reported outcomes. The questionnaires measured perceived knowledge, confidence, and preparedness, but did not measure objective competence. Therefore, the findings cannot demonstrate that students’ clinical performance or examination performance improved. Future evaluations should include objective outcomes such as OSCE station scores, written assessment performance, or directly observed practical skills assessments.

Second, the pre- and post-programme responses were anonymous and could not be matched at the individual level. As a result, the analysis treated responses as independent samples. This means that the study cannot confirm improvement within the same individual students, and the findings should be interpreted as group-level changes only.

Third, the post-programme sample size was smaller than the pre-programme sample. This introduces a risk of response bias because students who completed the post-programme questionnaire may have been more engaged with the teaching or more satisfied with the programme. This may have led to overestimation of post-programme acceptability and perceived preparedness.

Fourth, the mock OSCE questionnaire sample was small, with 10 pre-session and seven post-session responses. This limits the reliability and generalisability of the mock OSCE findings. The low response rate was partly related to the rapid five-week rotation structure, as students moved quickly between placements, and it was difficult to obtain follow-up responses once they had completed the block. These findings should therefore be interpreted as exploratory.

Fifth, the intervention changed over time in response to feedback. The early version included a quiz-style activity, whereas later versions placed greater emphasis on practical clinical examination skills. Although this reflects appropriate educational responsiveness, it also means that all cohorts did not experience the initial intervention identically. The results therefore reflect SPC Made Easy as an evolving teaching programme rather than a fixed intervention.

Finally, this was a single-centre evaluation conducted at one hospital site. The findings may not be directly generalisable to other institutions without adaptation to local curriculum requirements, staffing availability, student numbers, and placement structure.

## Conclusions

SPC Made Easy was associated with improved self-rated knowledge, confidence, and perceived preparedness among Year 4 medical students undertaking a SPC attachment. The programme provided structured, curriculum-mapped teaching across core surgical and peri-operative domains and was feasible to deliver within a five-week clinical rotation.

A key feature of the programme was its responsiveness to student feedback. Early feedback suggested that the quiz-style activity was less useful than practical, clinically relevant teaching. The programme was therefore modified to include practical examination skills such as digital rectal, breast, and testicular examination teaching. This strengthened the relevance of the intervention and aligned it more closely with students’ clinical and assessment needs.

These findings should be interpreted as improvements in perceived preparedness rather than evidence of improved clinical competence. The study was limited by small sample sizes, unmatched anonymous responses, a single-centre design, and reliance on self-reported outcomes. Future work should include matched longitudinal data, objective assessment outcomes, and a larger multi-site evaluation.

## Appendices

Appendix 1

Retrospective Year 5 Needs Assessment Questionnaire

The retrospective needs assessment was anonymous and used categorical and Likert-style response options. Seventeen Year 5 students responded. The questionnaire items were as follows:

1. How well organised was your surgical rotation?

2. How clear were the objectives and structure of the teaching sessions?

3. How confident were you in your understanding of surgical and peri-operative care before the programme?

4. How much did your understanding improve after the rotation?

5. By the end of the rotation, how confident did you feel in taking a surgical history?

6. By the end of the rotation, how confident did you feel in your understanding of relevant surgical anatomy?

7. By the end of the rotation, how confident did you feel in your understanding of the peri-operative care pathway?

8. By the end of the rotation, how confident did you feel in managing post-operative complications?

9. To what extent did the rotation help you feel more prepared for surgical placements or examinations?

10. Would you have benefited from a structured teaching programme?

Appendix 2 

Programme-Level Questionnaire Items

The pre-programme questionnaire assessed confidence in recognising and assessing common surgical conditions; knowledge of Surgery and Peri-operative Care (SPC) topics, relevant anatomy, anaesthetics, pre-operative care, ENT surgery, urological surgery, rheumatology, orthopaedic surgery, post-operative care, and complications; SPC OSCE confidence; and SPC MCQ confidence. It also asked students about their preferred learning styles, what they hoped to gain from SPC Made Easy, and the topics or skills in which they felt least confident.

The post-programme questionnaire assessed confidence in recognising and assessing common surgical conditions; knowledge of SPC topics and relevant anatomy; preparedness for Year 4 examinations and OSCEs; knowledge of anaesthetics, pre-operative care, ENT surgery, urological surgery, orthopaedic surgery, rheumatology, post-operative care, and complications; engagement with the weekly structure; willingness to recommend SPC Made Easy; perceived beneficial aspects; suggested improvements; remaining challenging topics; perceived impact on surgical clinical decision-making; confidence in OSCEs; and confidence in MCQs.

Appendix 3

Mock OSCE Questionnaire Items

The pre-mock OSCE questionnaire asked whether students had previously attended formal OSCE sessions, whether they had previously participated in peer-led or self-run OSCEs, the perceived effectiveness of any previous peer-led OSCE experience, their confidence in performing in an OSCE, their confidence in surgical-themed history-taking, their confidence in performing surgical-themed skills such as digital rectal examination (DRE) and testicular examination, their biggest concerns before the OSCE, their confidence in providing peer feedback and acting as an examiner, and what they hoped to gain from the mock OSCE.

The post-mock OSCE questionnaire assessed whether the session met the learning objectives; post-session confidence in OSCE performance, surgical history-taking, and surgical-themed skills; remaining concerns; scenario difficulty; organisation and clarity of instructions; helpful and less helpful station types; confidence in providing peer feedback and acting as an examiner; the usefulness of the examiner and feedback roles; willingness to recommend the session; and suggested improvements.
